# Platinum Nanoparticles Immobilized on Electrospun Membranes for Catalytic Oxidation of Volatile Organic Compounds

**DOI:** 10.3390/membranes13010110

**Published:** 2023-01-14

**Authors:** Karel Soukup, Pavel Topka, Jaroslav Kupčík, Olga Solcova

**Affiliations:** 1 Institute of Chemical Process Fundamentals of the CAS, CZ-165 00 Prague, Czech Republic; 2 FZU-Institute of Physics of the Czech Academy of Sciences, CZ-182 00 Prague, Czech Republic

**Keywords:** electrospun membranes, electrospinning, platinum catalysts, volatile organic compounds, polybenzimidazole

## Abstract

Structured catalytic membranes with high porosity and a low pressure drop are particularly suitable for industrial processes carried out at high space velocities. One of these processes is the catalytic total oxidation of volatile organic compounds, which is an economically feasible and environmentally friendly way of emission abatement. Noble metal catalysts are typically preferred due to high activity and stability. In this paper, the preparation of a thermally stable polybenzimidazole electrospun membrane, which can be used as a support for a platinum catalyst applicable in the total oxidation of volatile organic compounds, is reported for the first time. In contrast to commercial pelletized catalysts, high porosity of the membrane allowed for easy accessibility of the platinum active sites to the reactants and the catalytic bed exhibited a low pressure drop. We have shown that the preparation conditions can be tuned in order to obtain catalysts with a desired platinum particle size. In the gas-phase oxidation of ethanol, acetone, and toluene, the catalysts with Pt particle sizes 2.1 nm and 26 nm exhibited a lower catalytic activity than that with a Pt particle size of 12 nm. Catalysts with a Pt particle size of 2.1 nm and 12 nm were prepared by equilibrium adsorption, and the higher catalytic activity of the latter catalyst was ascribed to more reactive adsorbed oxygen species on larger Pt nanoparticles. On the other hand, the catalyst with a Pt particle size of 26 nm was prepared by a solvent evaporation method and contained less active polycrystalline platinum. Last but not least, the catalyst containing only 0.08 wt.% of platinum achieved high conversion (90%) of all the model volatile organic compounds at moderate temperatures (lower than 335 °C), which is important for reducing the costs of the abatement technology.

## 1. Introduction

Emission abatement has received extensive research during the last several decades and has become a public as well as political concern [1,2,3]. Volatile organic compounds (VOCs) represent one of the most important groups of gaseous pollutants. According to the generally used definition, VOCs involve organic compounds showing the normal boiling points in the range of 50–260 °C. They are detrimental to both human health (being toxic, carcinogenic, mutagenic) and the environment (being greenhouse gases and precursors of photochemical smog). Therefore, more stringent environmental regulations are being applied around the world in order to decrease the amount of VOC emissions. Catalytic oxidation is an economically feasible and environmentally friendly method for the abatement of VOCs t. Noble metal catalysts are often preferred due to their high activity and selectivity together with good stability and time-on-stream [4]. Designing catalysts with a low amount of active phase and a low-pressure drop is important for industrial applications [5,6,7].

Nowadays, nanostructures involving nanofibers, nanorods, nanotubes, and nanowires have a great application potential due to their unique properties (e.g., nanosize effect and interface effect, short transport lengths, or directional transmission) [8,9]. However, polymeric electrospun membranes reveal distinctive characteristics and superiority [10]. Nanofibrous membranes allow for the control of the specific surface area, catalyst accessibility [11] as well as the catalyst regeneration [12] from the reaction mixture for subsequent applications. Application of nanofibers as excellent supports is connected to the fact that a variety of polymers can be electrospun. They meet different requirements on supports; moreover, their high porosity and interconnectivity as the electrospun supports endow them with low mass transport resistance [13]. Smaller and larger molecules can diffuse surprisingly quickly in the amorphous and partially crystalline phase in polymer nanofibrous matrices in which the catalytically active agent is molecularly dispersed, both above and below the glass transition temperature.

Metal nanoparticles play an irreplaceable role in catalysis due to their high dispersion, large concentration of the highly under-coordinated surface sites, and the presence of the quantum confinement effects, which can improve their reactivity [14,15]. Nevertheless, the major difficulty regarding nanoparticles is their undesirable agglomeration to form larger particles. To prevent the formation of such agglomerates, surfactants [16], ligand exchange materials [17] and polymeric carriers [18] have been extensively applied. One of the new routes seems to be the deposition of nanoparticles directly on electrospun fibers from the solution [19,20,21,22]. However, polymeric nanofibers themselves do not, in general, show the quantum effects usually associated with nanoparticles. The reason lies in the fact that the nature of the electronic states of the organic compounds including polymeric materials, which can control both electronic and optical properties, does not correspond to the nature of semiconductors or conductors [23].

Polymeric nanofibrous membranes can be prepared by a number of techniques including a template synthesis, phase separation, self-assembly, or drawing [15,16]. Nevertheless, electrostatic spinning (electrospinning) currently represents not only the most straightforward but also the cheapest route for the production of nanofibers, even at an industrial scale [24]. The fiber formation during the electrospinning process is based on the electrical forces, and fiber forming takes place via a very peculiar self-organization process driven by the repulsive electrostatic forces [23].

The aim of this paper was to develop a method for the preparation of a nanofibrous polymeric membrane, which could be used as a support for noble metal nanoparticles. A polybenzimidazole electrospun membrane was prepared for the first time and employed as a support for platinum nanoparticles in order to create a new type of oxidation catalyst. Polybenzimidazole was selected due to its high thermal stability, and electrospinning was employed in order to prepare a polymeric support with morphology, which would be beneficial for its application in catalysis. Due to high porosity of the electrospun membrane and the resulting low mass transfer limitations, the catalytically active phase supported on the surface of the electrospun nanofibers would be readily accessible to the reactants. Moreover, high porosity of the support is important for the industrial application of the catalysts in order to achieve a sufficiently low pressure drop in the catalytic reactor. Herein, methods for the preparation of platinum nanoparticles supported on an electrospun polybenzimidazole membrane were developed, and the structure/composition–activity relationships in the oxidation of model VOCs (ethanol, acetone, toluene) were investigated.

## 2. Materials and Methods

### 2.1. Electrospun Membrane Preparation

**Figure 1:**
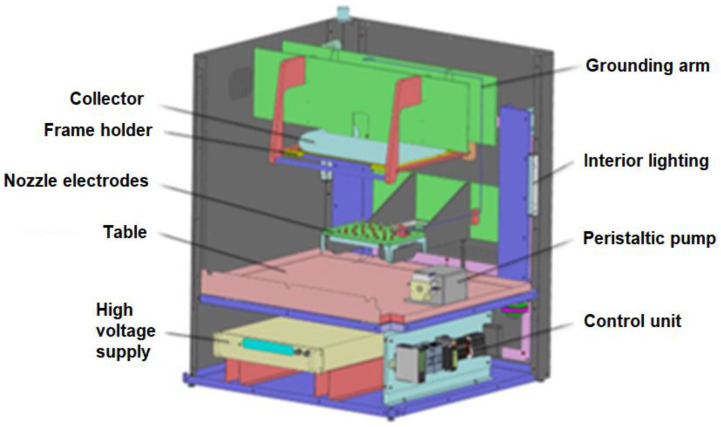
Setup of the electrospinning instrument used.

Polybenzimidazole (PBI), as a class of heat and chemicals extremely resistant polymers, was selected for the preparation of electrospun catalytic membranes. PBI was purchased from PBI Performance Products, Inc., Charlotte, NC, USA in the form of a solution (26 wt.%) in dimethylacetamide involving a stabilizer based on LiCl salt. Prior to the electrospinning process, the parent solution of PBI was diluted by a dimethylacetamide (≥99%, purchased from Sigma-Aldrich, St. Louis, MO, USA) solvent to 15 wt.%. Nanofibrous polybenzimidazole (PBI) membranes were prepared from a fresh solution by the electrospinning instrument (Cersum s.r.o., Liberec, Czech Republic) depicted in Figure 1. This setup consists of a steel multi-jet spinning electrode (24 nozzles) and a stainless-steel plate as the collecting electrode. The high voltage electrospinning electrode (multi-jet head) with +80 kV DC enabled the preparation of the nonwoven membrane with the width of 2 mm, revealing sufficient cohesion of individual nanofibers. The prepared electrospun membranes were dried in air (Elektrické pece Svoboda, Světice, Czech Republic) at 300 °C for 8 h to a constant weight. 

### 2.2. Electrospun Catalyst Preparation

**Figure 2:**
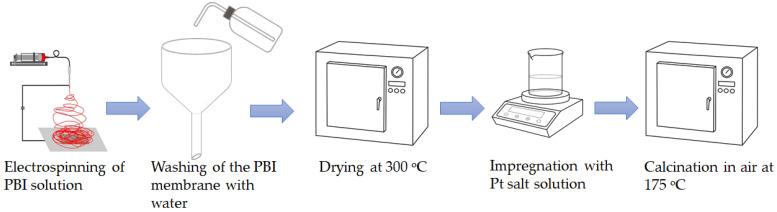
Overall scheme of the preparation of the electrospun catalysts.

Platinum nanoparticles were deposited on the parent PBI electrospun support by means of a wet impregnation route (see the overall scheme of the electrospun catalyst preparation in Figure 2). It is known that the dispersion of noble metal catalysts deposited on the surface of the polymeric membrane can be positively affected by the addition of citric acid [25], especially for low-temperature calcination.

Acetone (≥99.5% purchased from Sigma-Aldrich Co., St. Louis, MO, USA) and methanol (≥99.9% purchased from Fisher Scientific, Loughborough, UK) were chosen as appropriate indifferent solvents toward the parent polybenzimidazole support. Platinum(II) acetylacetonate (98%) was purchased from Stream Chemicals Inc. (Newburyport, MA, USA) and used as a precursor. The precursor was dissolved separately in the mixture of acetone and methanol (volumetric ratio 2:1). A total of 3.5 wt.% of citric acid (99.9%, purchased from Lach-Ner, s. r. o., Neratovice, Czech Republic) was added into the impregnation solution. The main reason was that citric acid improves the dispersion by serving as an anion and by creating a reducing atmosphere during the calcination.

The initial concentrations of metal salt as well as citric acid in the impregnation solution were in all cases 0.34 wt. % and 3.5 wt. %, respectively. Before the impregnation procedure, the PBI support was cut into 1–5 mm pieces that were washed with acetone and dried in static air at 150 °C for 12 h. Subsequently, the nanofibrous support was alternatively either immersed in the stirred activation solution with the stirring rate of 200 rpm and filtered out after 60 min of impregnation (the equilibrium adsorption method, used for the preparation of the 0.08 Pt/PBI and 0.54 Pt/PBI catalysts; the nominal Pt loadings were 1 and 9 wt.% of Pt, respectively) or placed into the rotary evaporator (Rotavapor R-300 Dynamic, BÜCHI Labortechnik AG, Flawil, Switzerland), and after 60 min of impregnation, the impregnation solution was evaporated (the solvent evaporation method, used for the preparation of the 0.68 Pt/PBI catalyst; the nominal Pt loading was 1 wt.% of Pt). The catalysts were dried in air at room temperature for approximately 3 h. After drying, the reduction by a heat treatment (calcination) was performed in a laboratory oven (Elektrické pece Svoboda, Czech Republic) at the temperature of 300 °C for 8 h. The catalysts were denoted as 0.08 Pt/PBI, 0.54 Pt/PBI, and 0.68 Pt/PBI according to the Pt content in the catalysts determined by atomic emission spectroscopy, which was found to be 0.08, 0.54, and 0.68 wt.% of platinum, respectively.

### 2.3. Electrospun Membranes and Catalyst Characterization

Nitrogen physisorption measurements at cryogenic conditions (77 K) were performed by an automated volumetric gas adsorption apparatus ASAP 2020 (Micromeritics, Norcross, GA, USA). To guarantee the accuracy of the obtained adsorption isotherms, highly pure nitrogen (99.9995 vol.%, purchased from Linde Gas Czech Republic, Prague, Czech Republic) as well as helium (99.9995 vol.%; Linde Gas Czech Republic, Prague, Czech Republic, used for determination of the free-space volume typically performed prior to analysis) were used. The high reliability of pressure measurements was achieved due to the three pressure transducers used, covering the working pressure from 0.13 mPa to 100 kPa. The equilibration time was set by several sets of the 15-fold repeated pressure measurements covering 10 s. The adsorption/desorption point was achieved and recorded only after the pressure change reached less than 0.01% between subsequent 15-fold pressure sets. Preceding the analysis, all samples were dried at 105 °C under a deep vacuum (<1 Pa) for 12 h. 

The specific surface area, *S_BET_*, was evaluated from the nitrogen adsorption isotherm in the range of relative pressure corresponding to *P*/*P^0^* = 0.05–0.25 using the standard Brunauer–Emmett–Teller (BET) procedure [26]. The mesopore surface area, *S_meso_*, and the micropore volume, *V_micro_*, were determined by the *t*-plot method [27]. The mesopore-size distribution was evaluated from the adsorption branch of the nitrogen adsorption–desorption isotherm using the Barrett–Joyner–Halenda (BJH) method via the Roberts algorithm [28,29]. The Harkins and Jura master isotherm [30] was used for the *t*-plot as well as for the mesopore-size distribution.

TEM analyses were performed on a FEI Tecnai F20 S/TEM (FEI Company, Hillsboro, OR, USA) with an X-TWIN lens, with a high brightness field emission electron gun (FEG) operated at 200 kV, equipped with a CCD camera (4 Mpix) for imaging in convectional mode and with a high angle annular dark field detector (HAADF) working in scanning (STEM) mode. The obtained electron micrographs were processed by a Digital Micrograph 3.20.1314.0 SW (Gatan, Pleasanton, CA, USA). Elemental analyses were performed with an energy-dispersive X-ray spectrometer (EDX) system (EDAX, Mahwah, NJ, USA) connected directly to the column of the microscope. EDX spectra were collected with a dispersion 20 ev/channel in the 0–40 keV range. TEM sample preparation: a small quantity of the given sample (which was a cluster of nanofibers covered with Pt nanoparticles) was placed on a glass slide and carefully disassembled using sharp tweezers. The obtained single fibers were carefully placed on standard TEM grids (covered with a carbon foil) that were pre-moistened with methanol. TEM grids were dried prior to the TEM analysis.

The morphology of the parent electrospun membranes was studied by a scanning electron microscope (TESCAN Vega III, Brno, Czech Republic). The membranes were sputtered with Au/Pt in plasma (approximately 5 nm) and analyzed for their fiber diameter and the pore-network topology. The image processing and image analysis were performed using the public domain ImageJ software (National Institutes of Health, Bethesda, MD, USA).

X-ray diffraction (XRD) measurements were carried out with a Bruker D8 Advance Eco diffractometer (Bruker Corporation, Billerica, MA, USA) equipped with Cu Kα radiation (λ = 1.54056 Å), and the signal was detected by a silicon-strip LynxEye detector whose energy resolution enabled us to eliminate fluorescence and Kβ radiation. Detailed data were collected in the 2ϴ range of 38–48°, step size of 0.02°. The qualitative analysis was performed using DIFFRAC.EVA software v5.1.0.5 containing the PDF2 database (2019 release). The full width in half-maximum (FWHM) values and crystallite sizes were evaluated using the same software. The crystallite sizes were evaluated using the Scherrer equation.

A FTIR spectrometer Thermo Nicolet Avatar 360 (Thermo Fisher Scientific Inc., Waltham, MA, USA) was used to measure the infrared spectra of the catalysts.

Surface elemental analyses were performed using a Kratos ESCA 3400 X-ray photoelectron spectrometer (XPS) (Shimadzu Corporation, Kyoto, Japan) and Mg Kα (1253.6 eV) was used for sample irradiation. The samples with the size of approximately 2 × 2 mm were placed on a carbon tape. The C, O, N, Pt, and Cl elements were detected and their most intense lines were measured in detail. The Shirley background was subtracted and elemental compositions of the layers were calculated from the corresponding areas. All of the spectra were corrected by shifting the main carbon C 1 s peak to 284.8 eV.

The prepared electrospun catalysts were thermally decomposed in the oven (Elektrické pece Svoboda, Czech Republic) at 750 °C for 6 h to determine the platinum loading on the electrospun membranes. Subsequently, the solid residues were dissolved in a mixture consisting of hydrogen peroxide and aqua regia. Finally, the prepared solution was assessed by microwave plasma atomic emission spectroscopy (MP-AES, Agilent Technologies, Inc., Santa Clara, CA, USA) equipped with an autosampler model SPS 3 (Agilent Technologies, Inc., USA). The sample was introduced by means of an inert OneNeb nebulizer with a double-pass glass cyclonic spray chamber system and a standard torch. The Agilent 4200 used magnetically coupled microwave energy to generate robust and stable plasma using nitrogen gas. The nitrogen was extracted directly from air from the environment using the nitrogen generator model 4107 (Agilent Technologies, Inc., USA), allowing for onsite testing in remote locations. 

### 2.4. Catalytic Experiments

The catalysts were tested in a fixed-bed reactor with an internal diameter of 5 mm. The catalytic tests of the total oxidation of volatile organic compounds (ethanol, toluene and acetone) were performed with 1000 ppm of individual VOC in air at the GHSV of 20 L g_cat_^−1^ h^−1^ using the temperature ramp of 2 °C min^−1^. The composition of the reaction mixture was analyzed by an Agilent 8890 gas chromatograph (Agilent Technologies, USA) coupled with a mass spectrometer. The *T*_50_ temperature, at which 50% VOC conversion was achieved, and the *T*_90_ temperature, at which 90% VOC conversion was achieved, were used to compare the performance of the catalysts. The catalytic activity was evaluated in terms of the turn-over frequency (TOF). The TOF was calculated in moles of converted ethanol, acetone, or toluene per mol of surface platinum atoms, and second at selected temperatures (167 °C, 225 °C, and 278 °C, respectively) employing the average Pt particle size determined by TEM (detailed information can be found elsewhere) [31].

## 3. Results and Discussion

### 3.1. Morphology

**Figure 3:**
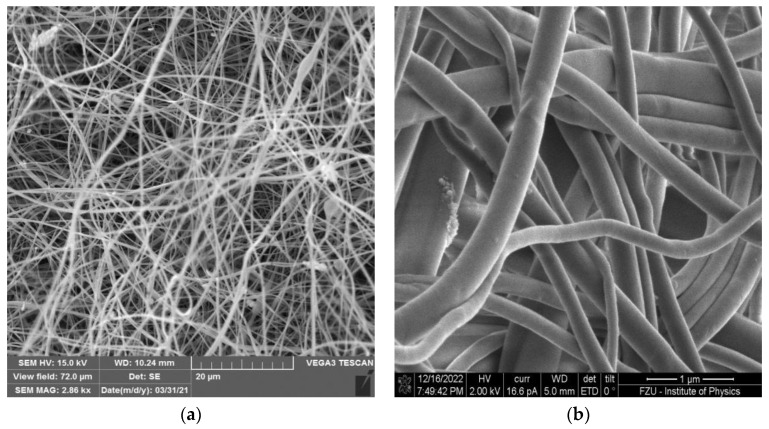
The SEM micrographs of PBI electrospun membrane at lower (**a**) and higher (**b**) magnification.

The assessment of the morphological properties of the prepared electrospun membranes were performed based on the corresponding SEM micrographs given in Figure 3.

The aim, from the morphological and microstructural point of view, was to prepare electrospun membranes revealing isotropic properties. It follows from our previous studies [13,19,31] that both the final morphology and effective transport properties of nanofibrous membranes mostly depend on the process conditions during the electrospinning process. However, the viscosity, which can be controlled directly by the concentration of polymeric solution, was recognized [22] as the most important parameter related to the microstructural (texture and effective transport) as well as morphological properties.

**Figure 4:**
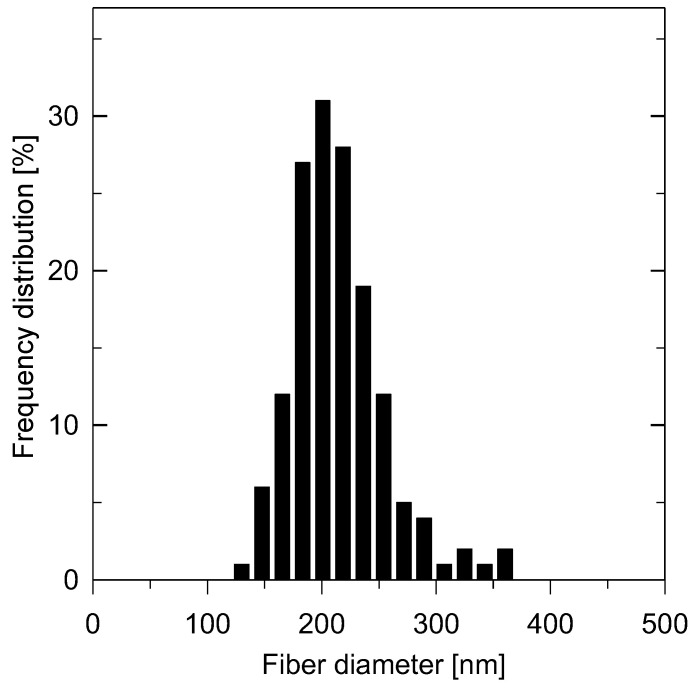
Distribution of nanofiber diameters in the parent electrospun membranes.

By means of the SEM micrograph given in Figure 3, it can be clearly seen that the electrospun membrane revealed good uniformity with predominantly bead-free fibers. As follows from most studies, electrospun nanofibers are being smooth, independently of the parent polymer used or process parameters [22], which was confirmed in Figure 3b. For the statistical calculation of the fiber diameter distribution, approximately 150 randomly distributed fibers over the SEM micrographs were taken into account. The calculated histogram of the PBI electrospun membrane is presented in Figure 4. It is evident that the prepared membrane consisted of nanometer-size fibers in the range from 130 to 360 nm with the maximum corresponding to 200 nm. 

### 3.2. Textural Properties

**Figure 5:**
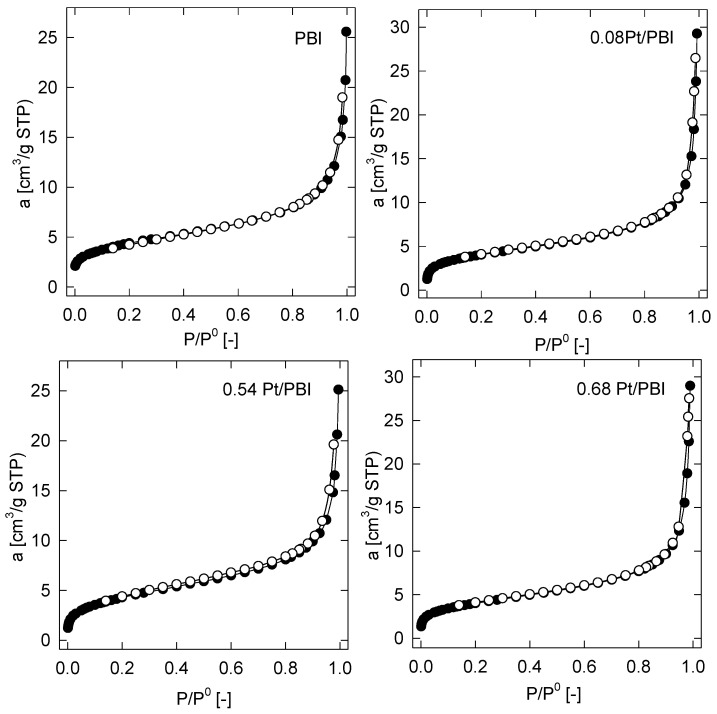
Adsorption (●) and desorption (o) branches of the nitrogen sorption isotherms at 77 K.

The basic textural properties involving the BET surface area (*S_BET_*), the specific surface area of mesopores (*S_meso_*), the specific total pore volume (*V_tot_*), and the specific volume of micropores (*V_micro_*) of both the parent electrospun membrane and nanofibrous catalysts are summarized in Table 1. It was found that the specific surface area of the parent electrospun membrane evaluated from the corresponding nitrogen adsorption isotherm by the BET approach (see Figure 5) achieved 16 m^2^/g. This confirmed that the impregnation method did not change the microstructural properties of the electrospun catalysts significantly, since they showed the same (0.54 Pt/PBI catalyst) or very similar (0.08 Pt/PBI and 0.68 Pt/PBI catalysts) magnitudes of the BES surface area as the parent membrane (PBI). The observed hysteresis loop between the adsorption and desorption branches of the sorption isotherms in Figure 5 is indicative of membrane mesoporosity (type IV isotherm according to IUPAC classification). Additionally, the corresponding specific surface area of the mesopores, *S_meso_*, was quantified by the *t*-plot method and are also given in Table 1. On the other hand, the presence of micropores was not confirmed in any of the samples under study. 

**Figure 6:**
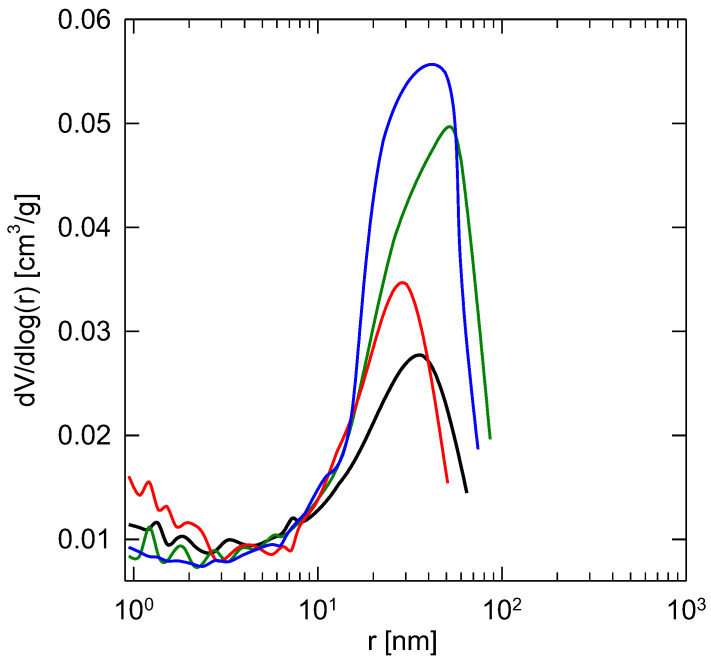
Pore-size distribution evaluated from the desorption branch of the nitrogen sorption isotherm at 77 K for the electrospun membrane (**—**) and catalysts (**—**): 0.08 Pt/PBI, (**—**): 0.54 Pt/PBI, (**—**): 0.68 Pt/PBI.

The pore-size distribution (PSD) functions of the electrospun membranes and catalysts are depicted in Figure 6. As can be seen, all tested samples revealed a unimodal distribution with the single highest value corresponding to the region of macropores (i.e., pores larger than 50 nm according to IUPAC definition [32]). The calculated maxima of a pore radius for the PBI membrane and 0.08 Pt/PBI, 0.54 Pt/PBI, 0.68 Pt/PBI catalysts were found to be 37, 45, 30, and 49 nm, respectively. These results confirm the very good agreement between the evaluated texture characteristics of the parent electrospun membrane and counterparts with Pt catalysts. The comprehensive texture results, together with the results from the SEM (Figure 3) and HRTEM analyses (Figure 6), indicate that no change in the microstructure of catalysts during their preparation and activation took place.

### 3.3. Physicochemical Properties of the Catalysts

**Figure 7:**
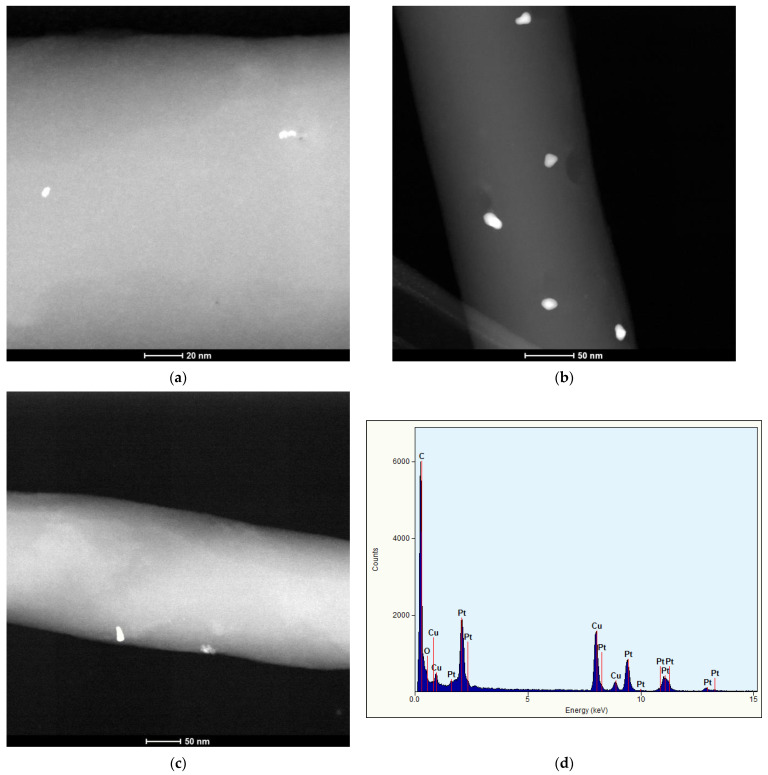
The STEM micrographs of the catalytic electrospun membranes. (**a**) 0.08 Pt/PBI, (**b**) 0.54 Pt/PBI, (**c**) 0.68 Pt/PBI, (**d**) typical EDX spectrum of an typical Pt NP (signal of Cu originates from the Cu TEM grid used).

The platinum content in the catalysts was determined by the MP-AES method as follows: the catalysts 0.08 Pt/PBI, 0.54 Pt/PBI, and 0.68 Pt/PBI contained 0.08, 0.54, and 0.68 wt.% of platinum, respectively. The corresponding mean sizes of the Pt nanocrystals were assessed by an image analysis of the STEM micrographs given in Figure 7. Based on the observation of the individual nanofibers (obtained from analyzed membranes) at higher magnification in HAADF-STEM mode, Pt nanoparticles were observable due to their higher Z-contrast as small bright objects on the grey bodies of PBI nanofibers (Figure 7a–c). The dimensions of the Pt nanoparticles were in units, max tens of nm (sizes are listed below). The presence of Pt was confirmed by the point EDX analysis performed using the analytical electron nanobeam focused directly on the analyzed nanoparticles. The typical EDX spectrum obtained from a Pt NP is depicted in Figure 7d.

The catalysts of 0.08 Pt/PBI, 0.54 Pt/PBI, and 0.68 Pt/PBI contained Pt nanoparticles with the mean size of 2.1, 26, and 12 nm, respectively.

**Figure 8:**
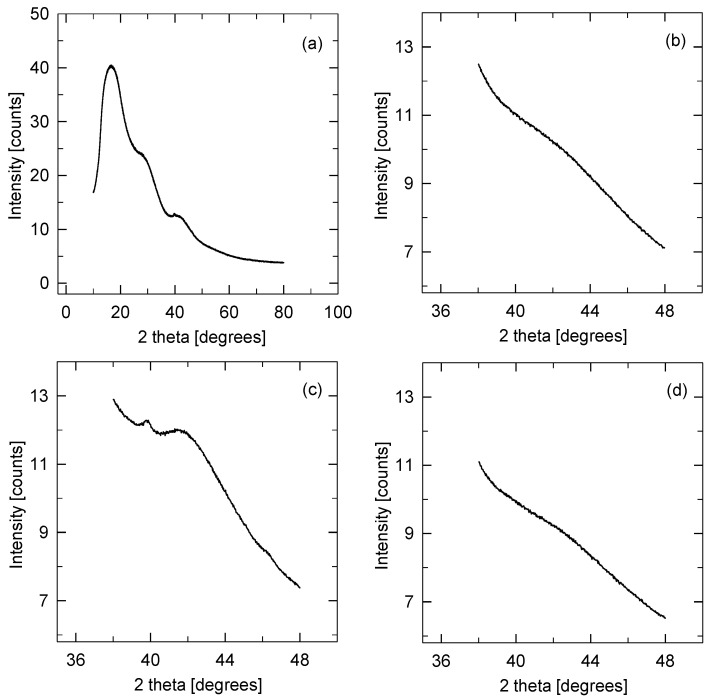
X-ray diffraction patterns of the sample: 0.54 Pt/PBI (**a**,**c**), 0.08 Pt/PBI (**b**), and 0.68 Pt/PBI (**d**).

The X-ray diffraction patterns of all catalysts (see Figure 8) mainly showed an amorphous structure with broad diffraction lines at 18° and 28° (2 theta), together with low and broad diffraction lines at 39°–46° (2 theta) that were ascribed to the polybenzimidazole structure. In the case of the 0.54 Pt/PBI catalyst, additional diffraction lines were observed at 39.7° and 46.2° (2 theta). These lines were ascribed to platinum with the fcc structure (PDF 00-004-0802), and the average Pt crystallite size of 25 nm was determined from the Scherrer equation. For other catalysts, no diffraction pattern of Pt was visible.

**Figure 9:**
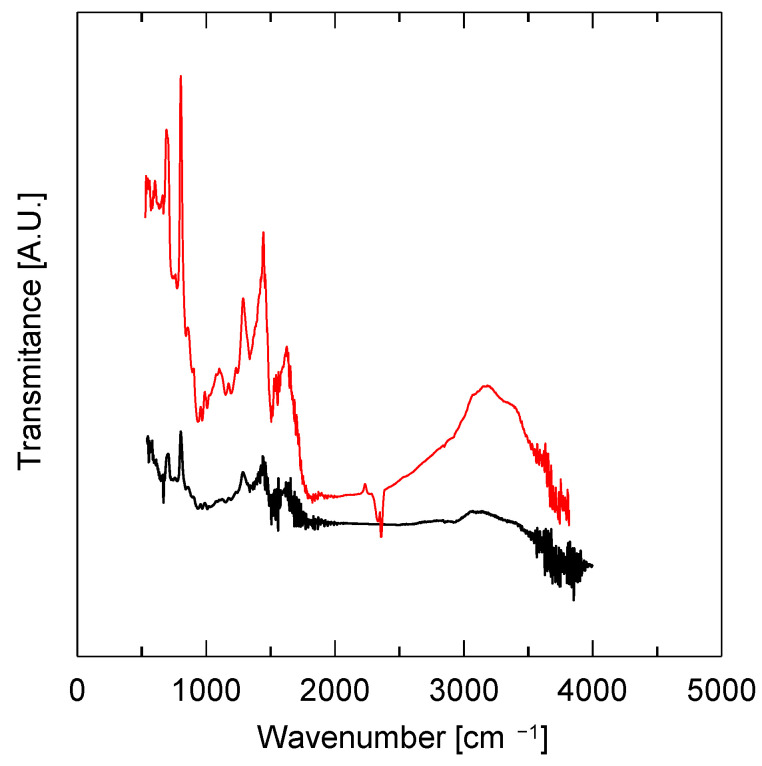
The FTIR spectra of electrospun membrane (**—**) and 0.54 Pt/PBI catalyst (**—**).

The FTIR spectra of the pristine PBI membrane and the 0.54 Pt/PBI catalyst are shown in Figure 9. Both spectra contain the bands typical for a PBI structure. Evidently, after impregnation of the electrospun membrane with platinum acetylacetonate and subsequent calcination, the structure of the polymer was preserved.

**Figure 10:**
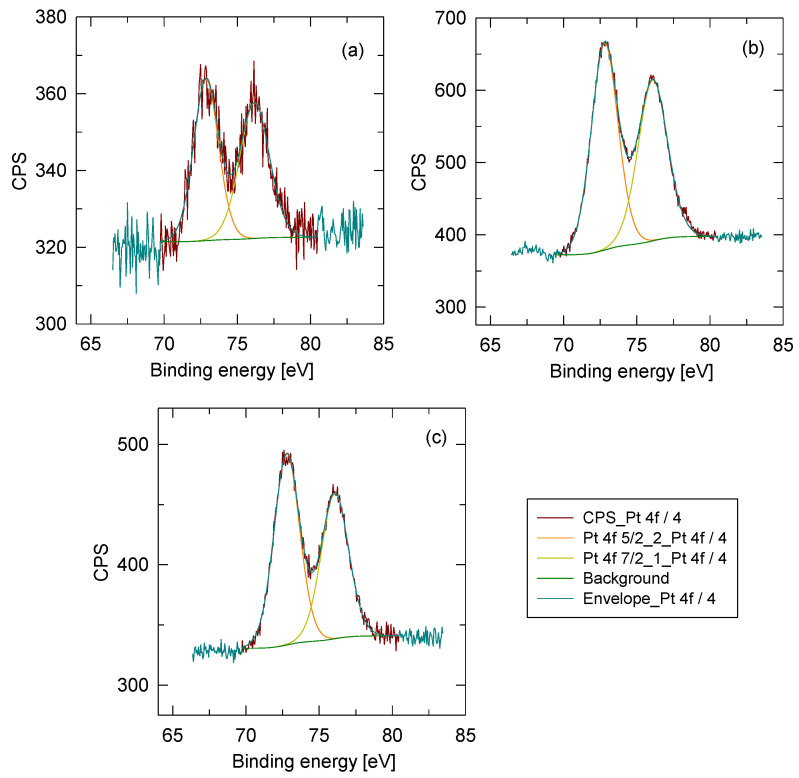
XPS spectra of the sample: (**a**) 0.08 Pt/PBI, (**b**) 0.54 Pt/PBI, (**c**) 0.68 Pt/PBI.

The XPS profiles of Pt are shown in Figure 10. However, the surface of all catalysts dominantly consisted of C, O, and N. The concentration of these three elements was consistent across all catalysts. In addition to these three elements, all catalysts also contained a small amount of Cl that originated from the polymer solution stabilizer (LiCl). The samples differed in Pt content. From the C 1s spectra, it could be seen that the nanofibers contained mainly C–C bonds (284.8 eV), with a small amount of C–O (286.4 eV) and C=O (289.1 eV) bonds. A part of the carbon and oxygen may have originated from the carbon tape used for the measurements. After the deconvolution of the N 1s spectra, the peak at 398.6 eV was ascribed to C=N–C, and the peak at 400.4 eV was ascribed to N-(C)3. No significant changes among catalysts were observed in the C 1s and N 1s spectra. In the Pt 4f spectra, a typical main peak of Pt 4f7/2 and Pt 4f5/2 with splitting at 3.32 eV was observed. The position of the main peak was 72.8 ± 0.1 eV and was ascribed to Pt(II). In the O 1s spectra, two peaks were used for deconvolution. The peak at 531.7 eV was ascribed to C=O, and the peak at 533.3 eV was ascribed to C–O. No oxygen peak was visible in the range from 529 to 530 eV, which could be ascribed to metal oxides.

### 3.4. Catalytic Tests

**Figure 11:**
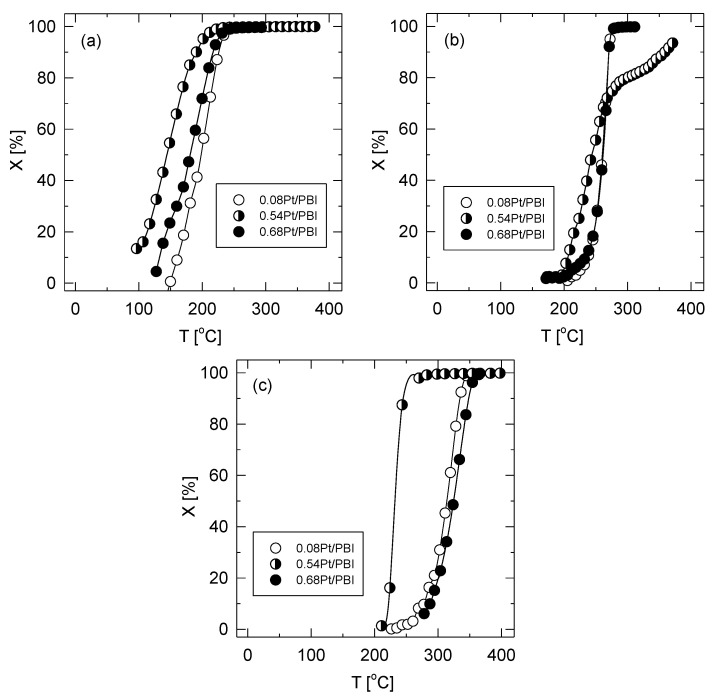
Conversion curves of ethanol (**a**), acetone (**b**), and toluene (**c**) over the investigated catalysts. Reaction conditions: 0.2 g of catalysts, 1000 ppm of VOC, GHSV 20 L g_cat_^−l^ h^−l^, temperature ramp 2 °C min^−l^.

The light-off curves of ethanol, acetone, and toluene oxidation over the investigated catalysts are shown in Figure 11. The performance of the catalysts was assessed according to the *T*_50_ and *T*_90_ values, whereas the activity of the catalysts was judged according to the turn-over frequency. The results are summarized in Table 2. The *T*_50_ temperature was used to compare the performance of the catalysts, as it is only affected to a limited extent by the mass and heat transfer limitations that may occur inside the catalyst particles. In our case, the *T*_50_ increased (i.e., the catalytic performance decreased) in the order 0.54 Pt/PBI > 0.68 Pt/PBI~0.54 Pt/PBI for all three model VOCs (ethanol, acetone, toluene). 

The aim of VOC abatement is the complete oxidation of a given VOC to carbon dioxide and water. Therefore, the *T*_90_ temperature was employed to illustrate the efficiency of the catalysts in the total oxidation. The *T*_90_ temperatures followed the same order as the *T*_50_ with the exception of acetone, where both 0.08 Pt/PBI and 0.68 Pt/PBI exhibited practically the same *T*_90_ temperatures (271 °C and 270 °C, respectively), while a higher temperature (359 °C) was needed to obtain 90% conversion of acetone over the 0.54 Pt/PBI catalyst.

**Figure 12:**
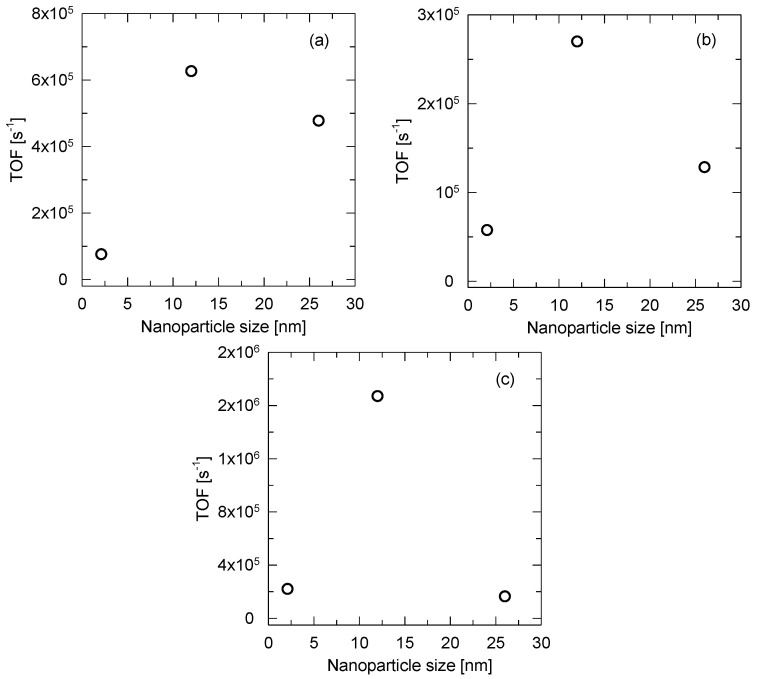
TOF depending on the mean Pt nanoparticle size for the total oxidation of (**a**) ethanol, (**b**) acetone, (**c**) toluene.

The turn-over frequency in moles of VOC per mol of surface platinum atoms, and second was calculated in order to evaluate the catalytic activity of the catalysts (Table 2). The dependence of the TOF on the mean Pt nanoparticle size determined by TEM is shown in Figure 12. In all cases, the 0.54 Pt/PBI catalyst was the most active, whereas the 0.08 Pt/PBI and 0.68 Pt/PBI catalysts exhibited a lower catalytic activity. It is well-known that the Pt dispersion is a major factor affecting the intrinsic activity in VOC oxidation, and that the reaction rates increase with increasing metal particle sizes [33]. This is due to the decrease in the Pt–O bond strength with the enlargement in the Pt particle size, which leads to more reactive adsorbed oxygen species on Pt sites [34]. Regarding this, the higher catalytic activity of the 0.54 Pt/PBI catalyst compared to the 0.08 Pt/PBI catalyst (both catalysts were prepared by the equilibrium adsorption method) can be rationalized. On the other hand, the 0.68 Pt/PBI catalyst was prepared using the solvent evaporation method. Consequently, a polycrystalline platinum was observed on the surface of this catalyst (while the TEM analysis revealed the mean Pt particle size of 26 nm, the XRD pattern of this catalyst did not contain the diffraction lines of platinum). Thus, the lower catalytic activity of this catalyst in comparison with the 0.54 Pt/PBI catalyst can be ascribed to the presence of polycrystalline platinum with coherent domains (grains) that were smaller than in the case of the 0.54 Pt/PBI catalyst (which exhibited the diffraction line of platinum in its XRD profile, and thus, the size of Pt coherent domains in the 0.68 Pt/PBI catalysts was apparently lower than 12 nm, which was the Pt particle size determined by TEM for the 0.54 Pt/PBI catalyst). Moreover, it was previously reported that different Pt grain types comparable to the single crystal surfaces Pt(100), Pt(110), and Pt(111) exhibited a different catalytic behavior in the oxidation reactions [35]. Thus, except for the size of coherent domains (grains) of platinum, their crystallographic modification may also play a role.

The above discussed results are in agreement with our previous study. We have reported the catalytic performance of platinum nanoparticles supported on poly(2,6-dimethyl-1,4-phenylene oxide) electrospun mats in VOC total oxidation [25]. We have shown that the *T*_50_ in methanol oxidation correlated with the mean size of the Pt nanoparticles, and the catalytic performance increased with the increasing Pt particle size from 2 to 8 nm. On the other hand, the main advantage of using the polybenzimidazole membrane as the catalyst support is its exceptional thermal stability compared to other polymers. While poly(2,6-dimethyl-1,4-phenylene oxide), which was employed as a polymeric electrospun support for noble metal oxidation catalysts for the first time [25], is thermally stable up to ~200 °C, the polybenzimidazole supported catalysts were stable up to ~350 °C and no deactivation of the catalysts was observed in the four consecutive light-off experiments.

## 4. Conclusions

A novel method for the preparation of a porous polybenzimidazole membrane by electrospinning and its utilization as a catalyst support for platinum nanoparticles was developed. The electrospun membrane was impregnated with platinum(II) acetylacetonate solution employing either equilibrium adsorption or solvent evaporation methods. The catalysts were tested in the oxidation of volatile organic compounds, which was employed as a model reaction.

The morphology of the membrane allowed us to prepare catalysts with high porosity and resulting low mass transfer limitations. Moreover, by tuning the preparation conditions, it was possible to obtain the catalysts with different platinum loading and nanoparticle size. The platinum active sites were deposited on the surface of the polymeric nanofibers. Thus, due to a high porosity of the membrane, they were readily accessible to the reactants. As a result, the catalyst containing only 0.08 wt.% of platinum was able to reach a high conversion (90%) of model volatile organic compounds (ethanol, acetone, toluene) at moderate temperatures (229 °C, 271 °C, and 334 °C, respectively).

Furthermore, we have shown that the catalytic activity, in terms of turn-over frequency, depends on the size and type of the platinum nanoparticles. The catalysts with Pt particle sizes of 2.1 nm and 26 nm exhibited a lower catalytic activity than that with a Pt particle size of 12 nm. The catalysts with a Pt particle size of 2.1 nm and 12 nm were prepared by the same method (equilibrium adsorption), and the higher catalytic activity of the latter catalyst can be explained by the decrease in the Pt–O bond strength with the enlargement in the Pt particle size, which leads to more reactive adsorbed oxygen species on the Pt sites. On the other hand, the catalyst with a Pt particle size of 26 nm was prepared by the solvent evaporation method and contained polycrystalline platinum. Thus, although the mean Pt particle size determined by transmission electron microscopy was 26 nm, the size of single coherent domains (grains) was much lower, which correlated with the lower catalytic activity of that catalyst.

In summary, a thermally stable electrospun polybenzimidazole membrane was prepared and for the first time employed as a support for platinum oxidation catalysts. The high performance of the 0.08 wt.% Pt catalyst is important for industrial applications as it enables both the investment costs (due to the low content of expensive noble metal) and the operating costs (due to the low reactor temperature that is needed to obtain full conversion of the pollutants) to be reduced.

## Figures and Tables

**Table 1:** Textural characteristics of the parent electrospun membrane and catalysts.

Sample	*S_BET_*	*S_meso_*	*V_tot_*	*V_micro_*
	[m^2^/g]	[m^2^/g]	(mm^3^_liq._/g)	(mm^3^_liq._/g)
PBI	16	14	26	<1
0.08 Pt/PBI	14	13	28	<1
0.54 Pt/PBI	16	16	25	<1
0.68 Pt/PBI	14	13	30	<1

**Table 2:** Temperatures *T*_50_ and *T*_90_ and turn-over frequency *TOF* in the oxidation of ethanol, acetone, and toluene. Reaction conditions: 0.2 g of catalyst, 1000 ppm of VOC, GHSV 20 L g_cat_^−l^ h^−l^, temperature ramp 2 °C min^−l^.

Catalyst	Ethanol			Acetone			Toluene		
	*T*_50_	*T*_90_	TOF*10^5^ (s^−1^)	*T*_50_	*T*_90_	TOF*10^5^ (s^−1^)	*T*_50_	*T*_90_	TOF*10^5^ (s^−1^)
0.08 Pt/PBI	203	229	0.5	260	271	0.4	314	334	1.6
0.54 Pt/PBI	144	190	6.3	244	359	2.7	234	251	16.7
0.68 Pt/PBI	181	217	4.8	261	270	1.3	325	349	1.7

## Data Availability

Not applicable.

## Abbreviations

*a*	Equilibrium adsorbed amount
BET	Brunauer–Emmett–Teller approach
BJH	Barrett–Joyner–Halenda
CCD	Charge-coupled device
CPS	Counts per second
DC	Direct current
EDX	Energy-dispersive X-ray spectrometer
FEG	Field emission electron gun
fcc	Face centered cubic
FTIR	Fourier transform infrared
GHSV	Gas hourly space velocity
HAADF	High angle annular dark field detector
HRTEM	High-resolution transmission spectroscopy
IUPAC	International Union of Pure and Applied Chemistry
liq.	Corresponding to liquid nitrogen
MP-AES	Microwave plasma atomic emission spectroscopy
*P*	Pressure
*P* ^0^	Vapor pressure
PBI	Polybenzimidazole
PSD	Pore-size distribution
*r*	Pore radius
*S_BET_*	The specific surface area
*S_meso_*	The specific area of mesopores
SEM	Scanning electron microscopy
STEM	Scanning transmission electron microscopy
STP	Standard temperature and pressure (273 K, 101.325 kPa)
*T*	Temperature
TEM	Transmission electron microscopy
TOF	Turn over frequency
*T*_50_	Temperature corresponding to 50% conversion
*T*_90_	Temperature corresponding to 90% conversion
*V*	Volume
VOC	Volatile organic compound
vol.	Volume
*V_micro_*	The specific volume of micropores
*V_tot_*	The specific total pore volume
wt.	Weight
X	Conversion
XPS	X-ray photoelectron spectroscopy
XRD	X-ray diffraction
